# CRISPR/Cas9 –based genome editing to expedite the genetic improvement of palms: challenges and prospects

**DOI:** 10.3389/fpls.2024.1385037

**Published:** 2024-03-13

**Authors:** SV Ramesh, MK Rajesh, Alpana Das, KB Hebbar

**Affiliations:** ^1^ ICAR-Central Plantation Crops Research Institute, Kasaragod, Kerala, India; ^2^ ICAR-Central Plantation Crops Research Institute Regional Station, Vittal, Dakshina Kannada, Karnataka, India; ^3^ ICAR-Central Plantation Crops Research Institute, Research Centre, Kahikuchi, Assam, India

**Keywords:** genomics assisted breeding, genome editing, palm improvement, palms, tree crops

## 1 Introduction

Palms encompass over 2,500 species, across 200 genera, ranking second only to grasses (Poaceae) and legumes (Fabaceae) in the realm of agricultural food production and industrial applications. The coconut (*Cocos nucifera* L.), arecanut (*Areca catechu* L.), oil palm (*Elaeis guineensis* Jacq.), and date palm (*Phoenix dactylifera* L.) are among the economically significant perennial species within the Arecaceae family. Coconut, often referred to as the “tree of life,” is celebrated for its diverse range of applications in food, nutrition, medicine, and various industrial uses (Ramesh et al., 2021). Coconut products encompass edible oil derived from the kernel or testa, tender coconut water, kernel, copra, coconut shell, coconut cake, wood-based products, coir pith, and items resulting from various valorization processes. The unopened spathe is tapped to extract inflorescence sap (*neera*), which can be further processed into jaggery, sugar, vinegar, and a variety of secondary products (Hebbar et al., 2022).

Arecanut (*Areca catechu* L.) is a crop in tropical Asia and certain parts of East Africa. In India, it holds a prominent place as a major commercial crop and is also medically important, primarily grown in a few states of the country. Nevertheless, its commercial products are distributed throughout India, and the country undeniably leads in terms of both area under cultivation and production, accounting for 54% of the world’s output. The fruit or nuts of the *Areca catechu* L. palm, commonly known as betel nut or *supari*, have a long history of use as a masticatory product by the Indian population, dating back to the Vedic period. As a result, arecanut is deeply intertwined with India’s history and social heritage. On a global scale, the betel quid is used by as many as 600 million people in Asia alone.

Date palm, on the other hand, thrives in arid regions such as Egypt, Iran, Saudi Arabia, and the UAE, among others (Aljohi et al., 2016). In addition to its fruit, date palm seeds also serve as a novel source of edible oil, further expanding its industrial applications (Ali et al., 2015). Oil palm stands out as an economically vital palm species, supplying approximately 35% of the world’s vegetable oil. The genetic improvement of oil palm could play a pivotal role in global nutritional security.

## 2 Palm problems

**Figure 1:**
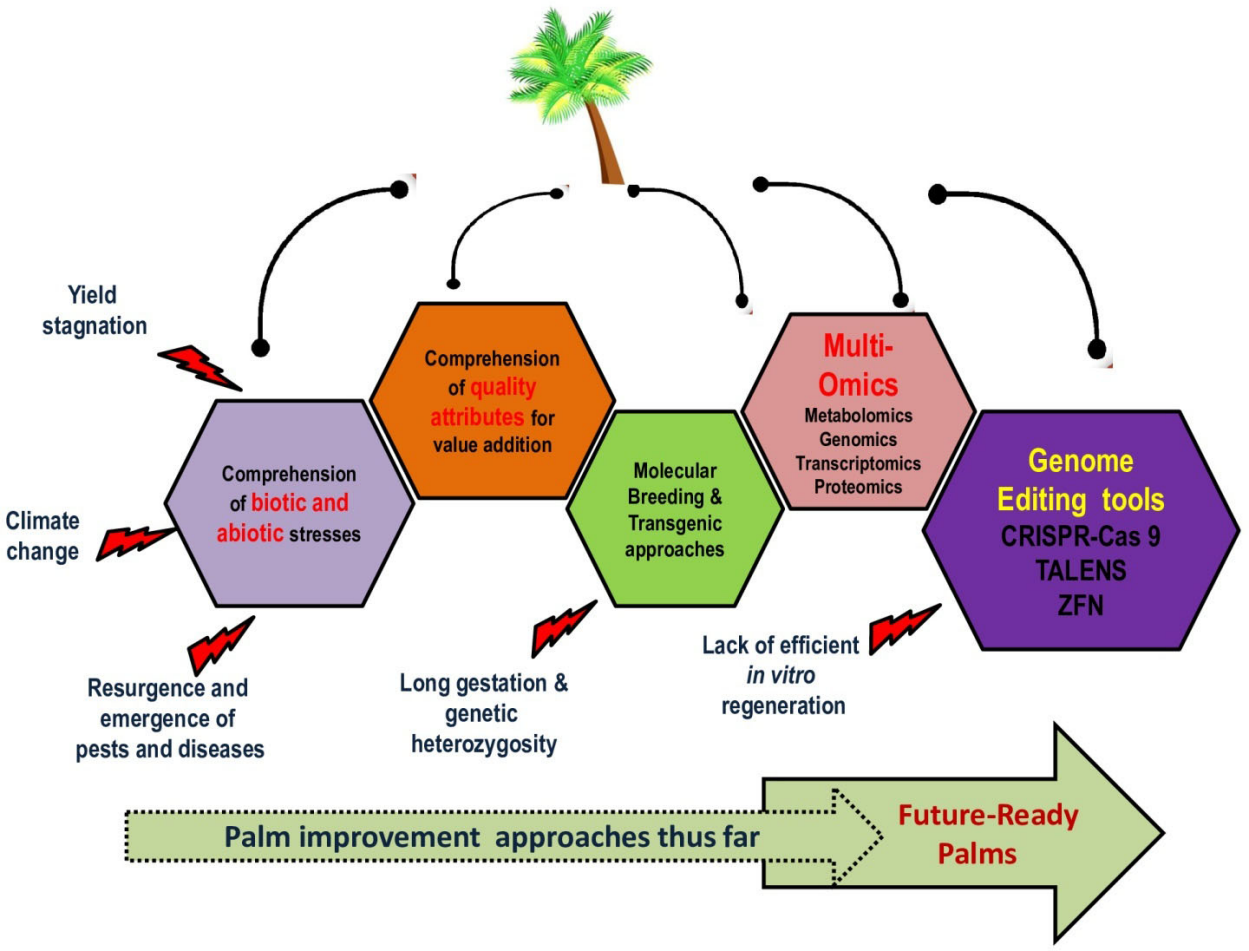
General considerations of research in palms and the defining role of genome editing in developing future-ready palms.

Economically important palm species worldwide are experiencing stagnation in yield, a rapid deceleration in land expansion, the effects of climate change, a surge in the cost of production due to agricultural inputs and labor, as well as biotic stressors like the emergence or reemergence of major pests and diseases, among other challenges (**Figure 1**). The long juvenile phase of palms, which can extend beyond 5-9 years, the time-consuming process of backcross breeding (30 years for date palms and 15-18 years for oil palms), and the reliance on seeds, as the primary propagule, severely hinder palm crop improvement programs. Therefore, expediting the adoption of novel breeding technologies, such as genome editing (GE), is imperative to ensure that missed opportunities in genomics-assisted breeding and genetic engineering, which have already revolutionized other cereal and legume crops, are not lost.

Genome editing (GE) approaches have been transforming the fields of plant breeding and genomics by enabling precise genetic manipulation of crop species. The utilization of CRISPR/Cas9 holds significant promise in revolutionizing agriculture and creating opportunities for innovative developments in plant gene editing systems (Chinnusamy et al., 2023; Saini et al., 2023). While the effectiveness of GE techniques has been demonstrated in other field crops and even in tree crops, it remains a relatively uncharted path for genetic improvement in palm species such as coconut, arecanut, or date palm, with the exception of oil palm. The utilization of genetic engineering technologies for commercial purposes in palms is currently quite limited. In addition to the complexity of palm genomes, significant obstacles related to genetic transformation and the development of efficient regeneration protocols pose major challenges to the widespread adoption of GE technologies.

## 3 Palm genomic resources

Continuous improvement of genomic resources for palm species is of paramount importance. Over the past decade, there has been a significant increase in the scale of genome assemblies for palms, opening the door to multi-omics studies. Genome assemblies are now available for economically important palm species such as oil palm (*Elaeis guineensis* Jacq), date palm (*Phoenix dactylifera* L), coconut (*Cocos nucifera* L), and arecanut (*Areca catechu* L), as well as related species like *Calamus simplicifolius* and *Daemonorops jenkinsiana*. These assemblies have been complemented by numerous resequencing and transcriptomics approaches (Al-Mssallem et al., 2013; Singh et al., 2013; Wang et al., 2021; Yang et al., 2021; Zhao et al., 2018). In this context, the development of the Arecaceae Multi-omics Database, ArecaceaeMDB (http://arecaceae-gdb.com), is a significant step forward. This database houses genomes of six economically important palms, along with resequencing data from 1631 different accessions, over 800 transcriptome sequences, and 138 metabolome datasets (Yang et al., 2023). However, unlike fruit and nut crops that benefit from well-assembled and high-quality genome sequences (Savadi et al., 2021), palms, such as date palm, present unique challenges due to their dioecious nature, requiring specialized assembly software and additional considerations for the development of effective genomic resources (Hazzouri et al., 2020). Nonetheless, the successful demonstration of the proof-of-concept of CRISPR/Cas9 application in various fruit tree crops, including apple, cacao, coffee, citrus, grape, pomegranate, pear, and walnut (Savadi et al., 2021), as well as in some forest tree crops (Cao et al., 2022) and in palms (Yeap et al., 2021), suggests that this approach may become a mainstay in the long run.

## 4 Potential applications of CRISPR-Cas9 system in palms

In the context of coconut, the identification of disease susceptibility factors represents a crucial area of research. This is essential to identify genomic regions or genes responsible for disease susceptibility, with the aim of manipulating them through the adoption of gene-editing technologies. Some of the potential gene targets include PR1, PR4, the pathogenesis-related genes transcriptional activator PT15-like gene, thaumatin-like protein, HSP70, and glutathione S-transferase. These genes have been identified as susceptibility factors in the case of root (wilt) disease in coconut (Rajesh et al., 2015; Verma et al., 2017; Arumugam and Hatta, 2022). Considering the diversity of pests affecting palms, adoption of multi-pronged strategies such as CRISPR-Cas9 based sterile insect technique, and targeting female insect reproductive fitness (eg., egg-specific protein encoding gene), or incorporating ovary targeting molecular signals in CRISPR-Cas9 system for heritable genome editing are warranted. These strategies aim to enhance the resilience of palm crops against insect pest damage.

In certain plants characterized by a low transformation efficiency, such as maize, the issue of limited transformation success has been successfully addressed through the over-expression of key regulators of somatic embryogenesis, such as Baby Boom (*Bbm*) and Wuschel2 (*Wus2*). While this particular approach has not yet been explored in the context of coconut, there is potential value in enhancing the regeneration process by adopting this method.

Palm products, especially those derived from the minimal processing of tender coconut nuts, require suitable anti-browning agents to prevent enzymatic browning reactions, ensuring visually appealing end products. In addition to the manipulation of agronomic traits, the application of the CRISPR/Cas9 system has been explored to mitigate enzymatic browning. This is achieved by creating mutations in the *StPPO*2 gene, resulting in a significant reduction in enzymatic browning by up to 75% (González et al., 2020). Similarly, Maioli et al. (2020) discussed the potential application of genome editing technologies in the development of eggplant berries, which exhibited a 52% reduction in PPO activity in edited lines compared to wild types, achieved by knocking out three PPO genes (*SmelPPO*4, *SmelPPO*5, and *SmelPPO*6). These studies demonstrate that, in addition to reducing enzymatic browning, the nutritional potential of post-harvest produce, including their antioxidant potential and phenolic content, is effectively preserved.

Palms in field conditions face a range of abiotic stresses induced by climate change, including monsoon variability, elevated temperature stress, rising atmospheric CO_2_ levels, and sea-level rise leading to salinity stress. Unlike annual crops, palms endure these abiotic challenges for an extended period during their lifespan, necessitating the adoption of genomics technologies to mitigate these stresses. Genetic mapping of genes associated with abiotic stress tolerance or related traits in mature palms is hindered due to their long juvenile phase and the costs involved in cultivating and maintaining large mapping populations in the field. Additionally, regions where palms are grown are experiencing increased soil salinity and sea-level rise, making it imperative to molecularly characterize abiotic stress response pathways.

Consequently, the adoption of CRISPR/Cas9 editing in palms involves numerous strategic considerations, including the selection of target genes, the sequence features of sgRNA, the method and tissue used for delivery, and the implementation of appropriate *in vitro* regeneration protocols. The highly heterozygous nature of palm genomes presents a challenge in designing sgRNAs that effectively match the target gene sequences near a protospacer adjacent motif (PAM) site. Traditional breeding methods, which can take 15-20 years or more, coupled with obstacles in developing genetic engineering-based high oleic acid-producing oil palm lines, have prompted the use of a multiplex CRISPR/Cas9 system to target multiple genomic sites (Bahariah et al., 2023). Given the nutritional significance of dietary oils, the application of CRISPR/Cas9 technology for targeting oil palm genes such as fatty acid desaturase 2 (*FAD2*) and palmitoyl-acyl carrier protein thioesterase (PAT) to modulate fatty acid metabolism and produce high-oleic acid oil holds significant promise for similar interventions in coconut palms (Bahariah et al., 2023).

Tree crops necessitate rapid genetic modification protocols and the development of genetically modified plants within a few generations as expediently as possible. In this context, the biallelic edits achievable through the CRISPR/Cas9 approach offer a means to swiftly generate genetically homozygous lines, circumventing the need for elaborate breeding methods to introduce homozygosity (Hazzouri et al., 2020). The successful establishment of electroporation-mediated protoplast transformation of sgRNA and genome editing components in oil palm suggests the potential for generating DNA-free genome-edited palms. However, the lack of an effective regeneration protocol for oil palm protoplasts significantly hampers the development of DNA-free genome-edited palms. Moreover, the creation of an efficient *in vitro* electro-transfection assay in oil palm for rapid assessment and evaluation of gRNA efficiency would substantially reduce the time and cost of transformation and regeneration, particularly for oil palm and other palm species (Yeap et al., 2021). In addition, development of gene editing protocol through *de novo* induction of meristems in dicots and the efficiency of cut–dip–budding (CDB) delivery system could enable rapid production of genetically modified germplasm (Maher et al., 2020; Cao et al., 2023). By implementing the PEG-mediated delivery system for Cas proteins and sgRNA, coupled with the establishment of a proficient protoplast regeneration system for palms, as successfully demonstrated in *Hevea brasiliensis* (Fan et al., 2020), we can significantly expedite the genome-editing-mediated breeding process.

Palm genomes are characterized by high allelic heterozygosity due to their outcrossing nature. Consequently, the presence of a high number of single nucleotide polymorphisms (SNPs) in the genome makes GE technologies less efficient in these crops. Thus, resequencing cultivars of interest and incorporating features related to the multi-ploidy nature of palm genomes into web-based algorithms, for sgRNA design in plants, are recommended to enhance the existing genomic resources for homozygous crops and model plants (Sattar et al., 2017). Nevertheless, it is anticipated that the application of CRISPR/Cas9-based genome editing will greatly facilitate the exploration of gene-function relationships and their impact on phenotypic traits, expediting crop improvement programs in palm species (**Table 1**).

**Table 1:** Major achievements in genome editing (GE) for tree crops and the prospects for its application in palms.

Sl. No	Species	Target gene(s)	Strategy	Features	Reference
1	Cocoa	*TcNPR3* (Non-Expressor of Pathogenesis-Related 3)	CRISPR/Cas9	Knock down of defense suppressor TcNPR3 resulted in enhanced resistance to pathogen *Phytophthora tropicalis*	Fister et al., 2018
2	*Elaeis guinensis*	*EgPDS* (phytoene desaturase) and *EgBRI1* (brassinosteroid-insensitive 1)	CRISPR/Cas9	Electroporation based transformation of protoplast showed 62.5-83.33% and 58.82-100% mutation frequency	Yeap et al., 2021
3	Sweet orange	*CsPDS* (phytoene desaturase)	CRISPR/Cas9 sgRNA	Agrofiltration utilizing Xcc targeting carotenoids biosynthesis caused 3.2-3.9% mutation but no albinos	Jia and Wang, 2014
4	Apple	Reporter gene *uidA*	ZFN (Zinc finger nuclease)	Stable and heritable gene mutation	Peer et al., 2015
5	*Populus tomentosa*	*PtoPDS* (phytoene desaturase)	CRISPR/Cas9	Mutation efficiency of 51.7% and albino phenotypes	Fan et al., 2015
6	*Hevea brasiliensis*	flowering time related genes (*HbFT1, HbFT2 and HbTFL1−1, HbTFL1−2, HbTFL1−3)*	CRISPR/Cas9	Used endogenous U6 promoters in protoplasts	Dai et al., 2021
7		*HbPDS*(phytoene desaturase)	CRISPR/Cas9	Stable transformation	Dai et al., 2021
8	*Eucalyptus grandis*	*CCR1* (cinnamoyl-CoA Reductase1)*, IAA9A* (an auxin-dependent transcription factor of Aux/IAA family)	CRISPR/Cas9	Wood-related genes edited in *Eucalyptus* hairy root	Dai et al., 2020
9	*Elaeis guinensis*	*EgPDS* (phytoene desaturase)	CRISPR/Cas9	Albino phenotypes with a mutation efficiency of 62.5–83.33%.	Yeap et al., 2021
		*EgBRI*1 (brassinosteroid-insensitive 1)	CRISPR/Cas9	premature necrosis and stunted phenotype	Yeap et al., 2021
		*EgWRKY, DREB1, EgRBP42, EgEREBP and EgNAC*	Base editing	Abiotic stress tolerance	Yarra et al., 2020
10	*Phoenix dactylifera*	*Pdpcs* and *Pdmt*	–	Abiotic stress tolerance (Cd and Cr) resistance	Chaâbene et al., 2018
		*Pdpcs* and *Pdmt*	–	Abiotic stress tolerance (heavy metals)	Chaâbene et al., 2017
11	*Cocos nucifera*	*PTI5 *PR1, PR4, *pathogenesis-related genes transcriptional activator PT15-like gene, *thaumatin-like protein, HSP70, *glutathione S-transferase	–	Root (wilt) disease resistance	Verma et al., 2017
		NBS-LRR type resistant gene analogues	–	Root (wilt) disease resistance	Rajesh et al., 2015
12	*Elaeis guineensis*	Genes of agronomic importance (disease resistance, abiotic stress tolerance, dwarfness)	–	Ganoderma disease, abiotic stress tolerance, ease of harvesting traits such as dwarf, long stalk	Yeap et al., 2021
13	*Phoenix dactylifera*	Genetic markers for sex determination	–	Early determination of sex distinguishes dioecious palms	Sattar et al., 2017
		Resistance genes in SNP desert of date palm	–	Abiotic and biotic stress tolerance	Sattar et al., 2017

## 5 Concluding remarks

Thus far, the CRISPR/Cas9 technology has demonstrated its effectiveness in genome editing of trees. Genome modification has been successfully accomplished in oil palm, and numerous fruit and nut tree crops. Several genes within these tree or perennials have manipulated through genome editing, aimed at enhancing resistance to both biotic and abiotic stressors, manipulating flowering and fruit ripening times, improving plant growth attributes, and enhancing the flavor profiles of their fruits. Innovations have led to the development of modified enzymes, offering increased efficiency in genome editing. Additionally, new and improved systems for gene editing and simultaneous activation of transcription have emerged, which are pertinent to the creation of novel palm varieties with wider applications.

## Author contributions

SR: Conceptualization, Formal analysis, Investigation, Resources, Writing – original draft, Writing – review & editing. MR: Formal analysis, Writing – review & editing. KH: Formal analysis, Resources, Writing – review & editing. AD: Formal analysis, Funding acquisition, Writing – review & editing.
